# MicroRNAs in plant-parasitic nematodes: what are they and why should we care?

**DOI:** 10.2478/jofnem-2025-0041

**Published:** 2025-09-24

**Authors:** Dave T. Ste-Croix, Benjamin Mimee

**Affiliations:** Agriculture and Agri-Food Canada, Saint-Jean-sur-Richelieu Research and Development Centre, Saint-Jean-sur-Richelieu, QC J3B 3E6, Canada

**Keywords:** cross-kingdom interaction, genomics, miRNA, management, plant-parasitic nematode

## Abstract

Plant-parasitic nematodes (PPNs) establish intimate interactions with their host plants, leading to significant economic losses worldwide. The molecular mechanisms underlying parasitism are complex, requiring tight regulation of numerous genes. MicroRNAs (miRNAs), small non-coding RNAs, regulate gene expression at the post-transcriptional level by binding to target messenger RNAs. However, the diversity and functional roles of miRNAs in PPNs are only beginning to be uncovered. This review summarizes the current knowledge on the nature, biogenesis, functions, and trafficking of miRNAs in PPNs. Beyond advancing our understanding of gene regulation throughout the nematode life cycle and during parasitism, miRNA characterization holds significant promise for novel control strategies. Emerging evidence suggests that miRNAs may function across kingdoms, modulating gene expression in host plants during parasitic interactions. We highlight compelling examples from other pathosystems and discuss preliminary findings on miRNA-mediated communication between PPNs and their hosts. Finally, we provide an overview of the main computational tools and databases available for identifying and predicting miRNAs and their targets, aimed at supporting researchers interested in this emerging field.

## Introduction

Plant-parasitic nematodes (PPNs) pose a significant threat to global agriculture, resulting in substantial crop losses and affecting food security (Kantor et al., 2024). The mechanisms by which PPNs successfully infect plants, bypass their natural defenses, and divert nutritional resources to their advantage are numerous and complex. This involves the timely production of a diverse array of proteins necessary to complete all developmental stages, recognize host plants, degrade cell walls, and neutralize defense mechanisms (Eves-van den Akker, 2021). These processes require intricate regulation of multiple genes and cellular pathways (Pellegrin et al., 2025).

MicroRNAs (miRNAs) are small endogenous non-coding RNAs, 21–24 nucleotides in length, which can interact with messenger RNAs (mRNA) and reduce the production of the corresponding protein, primarily by interfering with translation and/or inducing mRNA degradation (O’Brien et al., 2018). Modulation of gene expression can also occur remotely, that is, between cells, and thus miRNAs have the potential to influence multiple tissues. miRNAs may play an important role in regulating gene families that encode secreted proteins interacting with the host plant at specific stages of parasitism. These proteins, known as effectors, are defined as molecules secreted into the plant that target key host structures (e.g., the cell wall) or functions (e.g., plant immunity) to facilitate infection, and they remain a central focus of research in plant-PPN interactions (Vieira and Gleason, 2019).

It was also recently hypothesized that miRNAs produced by nematodes could modulate plant genes in a mechanism termed cross-kingdom (or trans-kingdom) interactions (Ste-Croix et al., 2023). For example, PPNs could secrete miRNAs that target plant defense genes to inhibit them. Recent evidence also indicates that the reverse mechanism—plants producing miRNAs that inhibit genes (such as virulence factors) in pathogenic organisms—may contribute to plant defense (Wu et al., 2023). Despite differences between miRNA mechanisms in plants, animals, and fungi and apparent incompatibilities between these systems, evidence supporting these hypotheses is rapidly accumulating. This emerging field of cross-kingdom RNA communication represents a paradigm shift in our understanding of how organisms interact at the molecular level during parasitism and could potentially revolutionize approaches to crop protection against PPNs.

It is established that plants respond to pests, in part by producing miRNAs, which are involved in several processes, notably the formation of feeding sites and the activation of defense responses (Hewezi, 2020). Manipulation of the expression of these miRNA genes has demonstrated their impact on plant resistance (Rambani et al., 2020). These aspects have been the subject of review articles (Siddique and Grundler, 2018; Jaubert-Possamai et al., 2019) and will not be addressed in this review. However, little information still exists on the diversity of miRNAs in PPNs and how they might interact directly (cross-kingdom interaction) or indirectly (e.g., by controlling effector expression) in parasitism. This review aims to compile and analyze the pertinent references discussing the role of miRNAs in PPNs, focusing on their diversity, functions, and implications in plant-nematode interactions.

## Biogenesis and Functions of miRNA

**Figure 1:**
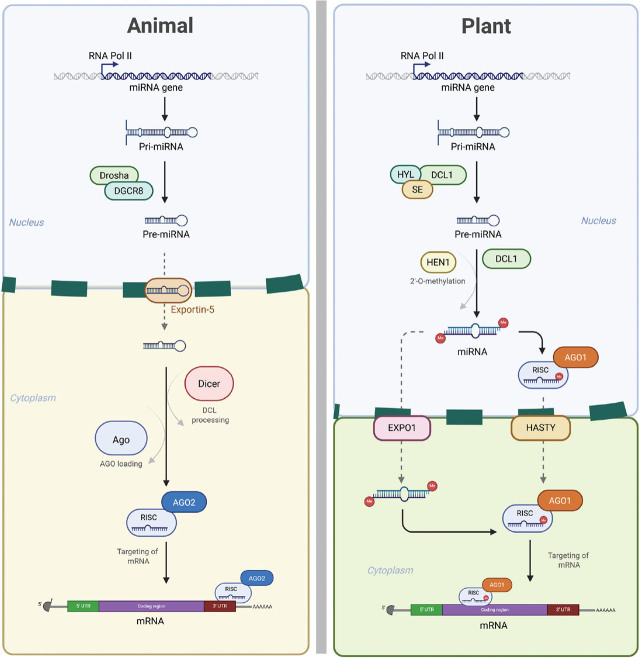
Canonical miRNA biogenesis pathways in animals and plants. miRNA synthesis begins with the transcription of pri-miRNAs, which are then cleaved into pre-miRNAs by different enzymes. These precursors are subsequently processed into mature miRNAs—within the nucleus in plants, but in the cytoplasm in animals. The resulting miRNA duplexes are then loaded onto different AGO proteins, with distinct AGO associations in plants versus animals. AGO, argonaute; miRNAs, microRNAs; pre-miRNAs, precursor miRNAs; pri-miRNAs, primary miRNAs.

While the topic of miRNA biogenesis has already been extensively reviewed (O’Brien et al., 2018; Wang et al., 2019), we here provide a brief overview of the key steps, highlighting the differences between plant and animal systems (Fig. 1). In both systems, miRNAs are encoded in the nuclear genome and can originate from various genomic regions, including introns of protein-coding genes, intergenic regions, and rarely in exons, typically of non-coding transcripts. They can be encoded as a single miRNA (MIR) gene or within co-regulated miRNA clusters (polycistronic MIR) located within *MIRNA* locus. It is worth noting that while intergenic MIR genes are transcribed under the control of their own promoters, intronic miRNAs are typically co-expressed with the host genes in which they reside. The biogenesis of mature miRNAs from these intronic miRNAs, also known as mirtrons, is generally considered part of a non-canonical pathway; a topic that will not be addressed in this section. In canonical miRNA biogenesis, the process begins with the transcription of primary miRNAs (pri-miRNAs), typically by RNA Polymerase II. These transcripts, usually around 1 kb in length, undergo a series of processing steps. In animals, the initial cleavage is carried out by the microprocessor complex, composed of Drosha and DGCR8 (also known as Pasha in invertebrates), which trims the pri-miRNA into a ∼70 bp hairpin-shaped precursor miRNA (pre-miRNA). While plants lack this microprocessor complex, a similar cleavage occurs through the activity of Dicer-like (DCL) proteins, which process the pri-miRNA into pre-miRNA of varying size and structure (Dong et al., 2008; Song et al., 2010). This is followed by further refinement by the same enzyme family, producing a mature miRNA duplex, composed of the guide- and passenger-strands, with characteristic 2-nucleotide overhangs. Within plants, these duplexes are generated within the nucleus and will undergo methylation of the 2′-OH position of their 3′ ends by HUAENHANCER1 (HEN1) protein to prevent degradation (Li et al., 2005). In animals, pre-miRNAs are exported to the cytoplasm via EXPORTIN5 prior to processing by Dicers into mature miRNA duplexes. Typically, one strand of this duplex is then selectively incorporated into an Argonaute (AGO) protein to form the RNA-induced silencing complex (RISC), which mediates sequence-specific gene regulation. As in animals, plant miRNA duplexes can be transported to the cytoplasm for AGO-loading by EXPORTIN5 homologs (Brioudes et al., 2021). However, emerging evidence suggests that AGO loading may also occur within the nucleus, with subsequent export of the AGO–miRNA complex (Bologna et al., 2018).

Post-transcriptional gene silencing (PTGS) occurs when miRNAs bind to specific regions of cognate mRNAs. However, the characteristics of these binding sites and the mechanisms of silencing differ substantially between plants and animals. In plants, miRNAs tend to interact within the protein-coding regions of mRNA, while in animals, these regions are predominantly located in the 3′ untranslated regions (3′UTR). Another key distinction between plant and animal miRNA pathways lies in the degree and nature of base pairing required between the miRNA and its target mRNA to trigger the silencing mechanism. In animals, perfect pairing within the miRNA seed region, typically spanning nucleotides 2–6, is often sufficient to trigger mRNA cleavage by AGO2, though most miRNA-target interactions in animals involve partial pairing that instead leads to translational repression (O’Brien et al., 2018). In contrast, plant miRNAs generally require near-perfect or perfect pairing across the entire length of the miRNA (Rogers and Chen, 2013). However, despite this high degree of complementarity, not all plant miRNA-target interactions result in mRNA degradation; translational inhibition can also occur depending on context and AGO association (Mallory and Vaucheret, 2010).

Post-transcriptional regulation enables the fine-tuning of gene expression, particularly for genes involved in key cellular functions such as development, differentiation, proliferation, and apoptosis. A notable example is *lin-4*, the first miRNA discovered in *Caenorhabditis elegans*, which was shown to control hypodermal cell fate during early larval development via interaction with the heterochronic gene *lin-14* (Lee et al., 1993; Wightman et al., 1993). Similarly, *let-7* and its related miRNAs (*mir-48*, *mir-84*, and *mir-241*) were found to also orchestrate the timing and fate of several developmental events through extensive interaction with the heterochronic genes *lin-14*, *lin-28*, *lin-41*, *lin-42*, and *daf-12* (Reinhart et al., 2000; Abbott et al., 2005). Although direct functional evidence of miRNA activity in PPNs remains limited, the conserved nature and roles of miRNA families suggest that these functions are likely preserved in these nematodes. This notion is supported by findings in *Heterodera glycines* (Ste-Croix et al., 2023), a sedentary endoparasite distantly related to *C. elegans*, where over half of the identified miRNAs (75/121) were shared with the latter. Insights into the developmental importance of miRNAs in *C. elegans* may therefore help inform their potential functions in PPNs. As miRNA function in *C. elegans* has already been comprehensively reviewed (Kotagama and McJunkin, 2024), it will not be revisited here.

While beyond the scope of this review, it is important to briefly acknowledge other classes of small non-coding RNA (ncRNA) that regulate gene expression and may be mistaken with miRNAs. Small interfering RNAs (siRNAs), for instance, are similar in size and operate via comparable gene-silencing mechanisms, yet they differ markedly in origin. Initially characterized in *C. elegans* (Fire et al., 1998), siRNAs are generally derived from exogenous double-stranded RNA sources, such as viral genomes or synthetic constructs, and are not encoded within the genome, distinguishing them from miRNAs (Table 1). However, these should not be confused with endogenous siRNAs (endo-siRNAs), which arise from internal cellular processes, often mediated by RNA-dependent RNA polymerase 6 (RDR6) and DCL enzymes, and function as part of an RNA silencing amplification mechanism in PTGS (Piatek and Werner, 2014). Among endo-siRNAs, several plant-specific subclasses have been identified, including trans-acting small interfering RNAs (tasiRNAs), phased secondary siRNAs (phasiRNAs), and epigenetically activated siRNAs (easiRNAs), each playing distinct roles in gene regulation and epigenetic control (Yoshikawa, 2013; Koch, 2014; Liu et al., 2020). Another class of similarly sized small non-coding RNAs is the intron-derived PIWI-interacting RNAs (piRNAs). First identified in *Drosophila* (Lin and Spradling, 1997), piRNAs associate with PIWI proteins to mediate the silencing of transposable elements, thus maintaining genomic integrity. More recent studies, however, suggest that piRNAs may also contribute to the regulation of gene expression beyond transposon control (Sun et al., 2022).

**Table 1:** Distinguishing characteristics inherent to miRNA and siRNA.

**Feature**	**miRNA**	**siRNA**
Origin	Endogenous	Endogenous and exogenous
Structure	Hairpin precursor	Long dsRNA
Processing enzymes	Dicer1 or DCL1 (in nucleus, mostly)	Dicer2 or DCL4 (also DCL2, DCL3 depending on siRNA class)
Target complementarity	Partial (often with central mismatches) or near perfect	Usually perfect or near-perfect
Function	Post-transcriptional gene regulation (mRNA degradation or translational inhibition)	Gene silencing, antiviral defense, transposon suppression, RdDM
Target specificity	Often targets multiple genes in the same family	Highly specific to one or few targets

DCL, dicer-like; dsRNA, double-stranded RNA; miRNA, microRNAs; mRNA, messenger RNA; RdDM, RNA-directed DNA methylation; siRNA, small interfering RNAs.

## miRNA Trafficking and Secretion

Recent studies highlight the emerging role of secreted endogenous miRNAs (ex-miRNA) in mediating cell-to-cell communication (reviewed in Chen et al., 2012; Zhao et al., 2019). While the full range of miRNA secretory pathways remains under investigation, two primary mechanisms are frequently reported: active secretion and passive leakage.

Passive leakage of miRNAs typically occurs when cells are injured, undergoing apoptosis, or when their plasma membranes become compromised. In these situations, miRNAs, alongside other cellular contents, can leak into the surrounding environment (Chen et al., 2012). Beyond this passive leakage, cells can also actively secrete miRNAs through extracellular vesicles (EVs), such as exosomes and cellular membrane-generated microvesicles. For instance, exosomes are formed inside cells through the inward budding of endosomal membranes, encapsulating miRNAs and protecting them from degradation while improving their stability in circulation (Chen et al., 2012; Zhang et al., 2015). The available evidence points toward these vesicles playing a key role in cell-to-cell communication by delivering miRNAs directly to recipient cells (Zhang et al., 2015; O’Brien et al., 2018). Interestingly, the packaging of miRNAs into these vesicles does not appear to be entirely random, as cells can selectively sort specific miRNAs (Chen et al., 2012; Zhao et al., 2019). While much about this process is still unclear, one well-characterized mechanism for miRNA sorting into EVs involves specific RNA-binding proteins (RBPs), including AGOs, that help selectively package these ncRNAs (Turchinovich et al., 2013; Santangelo et al., 2016). Another pathway for miRNA secretion involves microvesicle-free release, where miRNAs circulate bound to AGO protein complexes. In some studies, this microvesicle-free pathway has been reported to account for 90%–99% of all circulating miRNAs (Arroyo et al., 2011; Turchinovich et al., 2011). Albeit a very small proportion of circulating miRNAs, miRNAs have also been observed to be secreted alongside lipid bodies such as high-density lipoproteins (HDLs) and low-density lipoproteins (LDLs) (Wagner et al., 2013).

Upon reaching recipient cells, miRNAs will primarily gain access to the cytoplasm through endocytosis (Xu et al., 2013). In addition to endocytosis, miRNAs can also be delivered through direct membrane fusion of microvesicles, often facilitated by surface protein interactions, or may remain extracellular, where they interact with surface Toll-like receptors to trigger intracellular signaling pathways. (Fabbri et al., 2012; O’Brien et al., 2018).

### miRNA in PPNs

Since first being discovered in *C. elegans* in 1993 (Lee et al., 1993), miRNAs have now been described in many nematode species, including free-living, for example, *Pristionchus pacificus, C. briggsae, C. remanei* (de Wit et al., 2009), and parasitic species, for example. *Haemonchus contortus* (Marks et al., 2019), among many others. However, the characterization of miRNAs in PPN species is still in its early stages and is limited to a few species.

The first characterization of miRNA in PPN was in the pinewood nematode, *Bursaphelenchus xylophilus* (Huang et al., 2010). Although constrained by the methodologies available at the time, this study successfully identified and experimentally validated 57 miRNAs, shedding light on their evolutionary conservation across diverse eukaryotic lineages. Eighteen of these miRNAs were conserved across several animal groups, including *C. elegans*, *Drosophila melanogaster*, and humans, and a substantial proportion (38 out of 57) were shared between *B. xylophilus* and *C. elegans*, highlighting a high degree of conservation within nematodes. Another study on *B. xylophilus* expanded its known miRNAome (the complete set of miRNAs encoded in its genome) by identifying several novel miRNAs (Ding et al., 2015). The authors also correlated the expression of two miRNAs, predicted to interact with perfect seed region complementarity, with a glycoside hydrolase involved in plant cell wall degradation. They observed a mutually exclusive expression pattern, suggesting that this key infection step may be regulated by these miRNAs.

However, the most studied species of PPN for miRNAs is *Meloidogyne incognita*, with the first paper in 2015, which combined sequencing of small RNA-enriched libraries with predictive computational approaches (Wang et al., 2015). Through this study, the authors identified 102 mature miRNA sequences encoded within 71 distinct genes, of which 27 were homologous to known sequences in other nematodes. Several of these MIR genes were shown to be grouped into genomic clusters, an organization that has also been observed in several other organisms to date. A second study on *M. incognita* further expanded these numbers to 289 miRNAs, of which 35 were specific to this species (Zhang et al., 2016b). In both studies, significant differences in the expression profiles of these miRNAs were observed, with only a few being highly expressed. All of these highly expressed miRNAs were conserved across species and are likely essential in regulating nematode development. Additionally, significant differences in MIR gene expression between developmental stages were observed, and prediction of mRNA interactions revealed how these miRNAs could modulate specific metabolic pathways during the development of *M. incognita* (Subramanian et al., 2016; Kaur et al., 2017; Liu et al., 2019). Further emphasizing this importance, several miRNAs were found to be stage-specific, suggesting critical roles in developmental or infection processes. The comparison of miRNA expression with that of mRNAs predicted to be their targets in different stages has revealed probable regulatory networks for important functions (Mani et al., 2021).

Most recently, the soybean cyst nematode *H. glycines* became the latest PPN species to have its miRNAome characterized. In addition to identifying miRNA genes and quantifying their expression across all developmental stages, Ste-Croix et al. (2023) also compared the miRNAome of *H. glycines* to those of other PPN species. Among the newly identified miRNAs, they found that 75% were shared with *H. schachtii* (same genus), fewer than 10% with *Globodera rostochiensis* and *G. pallida* (same subfamily), and none with *Meloidogyne* species (same family). Based on estimated divergence times among these groups, this corresponds to a miRNA birth rate comparable to that of other animals, but substantially higher than in *C. elegans*. Predicting the mRNA targets within the nematode revealed that, on average, each nematode gene was predicted to interact with three different miRNAs, while each miRNA was predicted to target hundreds of genes. Although these numbers varied greatly between individual miRNAs and are likely overestimated due to the nature of computational predictions, they illustrate the extensive regulatory potential of miRNA-mediated gene expression. Furthermore, analysis of miRNA-effector interactions revealed that effectors were primarily predicted to interact with species-specific miRNAs, suggesting recent evolutionary origins and implication in species-specific traits (e.g., host range). Interestingly, a few unique miRNAs were predicted to interact with several effectors and may serve as general modulators of their expression, similar to what was recently observed with transcription factors (Pellegrin et al., 2025).

The involvement of miRNAs in regulating specific effectors has been directly demonstrated in *M. graminicola*. Using a miRNA target prediction algorithm (see Section “miRNA Discovery and mRNA Target Prediction in PPN”), followed by validation through an *in vitro* dual-luciferase reporter assay, it was shown that mgr-miR-9 modulates the expression of the protein disulfide isomerase MgPDI (Tian et al., 2023), while mgr-miR-228 regulates the expression of the transthyretin-like protein MgTTL1 (Tian et al., 2025). In both cases, repression of these miRNAs during infection resulted in upregulation of their respective effector genes. Furthermore, the application of synthetic miRNAs led to downregulation of the target effectors, and subsequent bioassays revealed a significant reduction in the reproductive capacity of *M. graminicola*. These findings support the idea that miRNAs may play a critical role in the fine-tuned and dynamic regulation of gene expression throughout the different stages of infection.

Moreover, miRNA activity may potentially extend to the regulation of host plant genes as well. Exosomes (microvesicles involved in the transport of small molecules) were also isolated from *H. glycines* and were shown to contain miRNAs, some of which show strong homology to host genes (Ste-Croix et al., 2023). While these predictions remain exploratory due to the fundamental differences in gene regulation between plants and nematodes, they raise a compelling hypothesis about cross-kingdom regulatory interactions in PPN.

### Cross-Kingdom Interaction

RNA, in all its forms, is commonly found in the environment but is typically degraded rapidly. The discovery of stable plant-derived miRNAs circulating in the serum of humans and animals was unexpected (Zhang et al., 2012). These miRNAs, originating from food intake, were not only capable of being absorbed but also exhibited resistance to degradation by RNases and digestive enzymes. Even more surprisingly, plant miRNAs were shown to suppress gene expression in the animal host, mimicking the regulatory function of endogenous eukaryotic miRNAs. Subsequently, it was demonstrated that parasites, such as the gastrointestinal nematode *Heligmosomoides polygyrus*, can also secrete miRNAs that are detectable in host tissues and modulate the immune response (Buck et al., 2014). Purified exosomes containing nematode-derived miRNAs, and in some cases even the nematode AGO protein, were sufficient to suppress host immunity upon injection, in the absence of the nematode itself. In contrast, the mechanisms of miRNA action in plants differ substantially from those in animals, raising the question of whether eukaryotic parasites that infect plants might similarly manipulate host gene expression through the release of miRNAs.

One of the first examples of cross-kingdom short RNA (sRNA) interactions in a plant pathosystem was found in the causative agent of the gray mold disease, *Botrytis cinerea.* The fungus has been shown to suppress the immune response in tomato by binding to the plant’s AGO proteins and specifically inhibiting the expression of MAP kinases involved in this defense pathway (Weiberg et al., 2013). Prior to this report, most of the known virulence factors or effectors were of protein origin. However, this study confirmed that sRNAs could also function as effector molecules and may represent key elements of fungal pathogenicity. To reach its targets within the plant cells, the fungus was shown to employ EVs containing sRNAs, which were secreted and subsequently internalized by plant cells via clathrin-mediated endocytosis (He et al., 2023). Interestingly, the reverse was also shown to be true with a tomato-derived miRNA inhibiting the virulence of *B. cinerea* (Meng et al., 2020). Indeed, bioassays demonstrated that, following infection, the expression of the tomato miR396a-5p was upregulated, while the expression of confirmed fungal virulence genes was subsequently repressed (Wu et al., 2023). Moreover, exogenous application of this miRNA was sufficient to suppress fungal virulence. A similar mechanism has also been observed in the interaction between *Verticillium dahliae* with cotton, as well as within other pathosystems (Zhang et al., 2016a; Wang et al., 2017). While evidence and review papers on cross-kingdom interactions are accumulating (Liang et al., 2013; Wang et al., 2017, 2018; Zhou et al., 2017; Gualtieri et al., 2020; Chowdhury et al., 2024), there are still few examples in PPNs.

Comparative analysis of *H. glycines* miRNA sequences with those of its host plant predicted 1,542 potential interactions involving 1,281 unique plant genes (Ste-Croix et al., 2023). When focusing only on miRNAs also detected in nematode-derived exosomes, thus more likely to reach the cytoplasm of plant cells, 540 interactions involving 482 genes remained. Several of these target genes were directly involved in the plant’s defense response against the nematode, supporting the hypothesis that *H. glycines* may secrete miRNAs as effector molecules to manipulate host gene expression to its advantage. This remains a speculative hypothesis, as multiple barriers must be overcome for these miRNAs to be functional in the host. Specifically, the miRNAs must be expressed at the appropriate time, packaged into exosomes (or secreted via other mechanisms), delivered into plant cells, transported to the cytoplasm, and be compatible with the plant’s AGO proteins and RISC complex. Nonetheless, the possibility of cross-kingdom gene regulation via nematode-secreted miRNAs represents a compelling area for further investigation. Interestingly, in the same study, nine miRNAs were predicted to target both nematode effector genes and host genes located near known soybean resistance loci. These miRNAs were specific either to *H. glycines* (*n* = 4) or to the *Heterodera* genus (*n* = 5), suggesting they may have evolved relatively recently in association with the obligate parasitism and host specialization of these nematodes.

A similar study was conducted in the tomato-root-knot nematode pathosystem. Based on sequence complementarity, bioinformatic predictions identified 523 putative cross-kingdom interactions, involving 105 *M. incognita* miRNAs predicted to target 469 tomato genes (Leonetti et al., 2024). In contrast, thousands of interactions were predicted in the opposite direction, with tomato-derived miRNAs potentially targeting *M. incognita* genes. Functional enrichment analysis of the highest-confidence interactions revealed that the tomato genes targeted by nematode miRNAs are primarily involved in plant development and stress responses. Conversely, plant miRNAs were predicted to influence a wide range of nematode processes, including mobility, host recognition, feeding behavior, and overall development. As noted earlier, these predictions remain speculative. However, in this case, the authors went a step further by experimentally validating a subset of the predicted interactions. *M. incognita* second-stage juveniles (J2s) were soaked in synthetic tomato miRNAs predicted to have cross-kingdom targets. This treatment led to a significant reduction in the expression of two target genes (Minc11367 and Minc00111). In planta bioassays further demonstrated a marked reduction in root swelling and gall formation in nematodes pre-treated with two specific tomato miRNAs (sly-miR156a and sly-miR169f), compared to untreated nematodes (Leonetti et al., 2024).

These studies indicate that it is possible for plants and their nematode pathogens to exchange miRNAs, often transported via EVs, and that these sRNA effectors play roles in both defense and virulence (Cai et al., 2021). This opens the door to novel control strategies that could exploit this molecular cross-talk for targeted pest and pathogen management.

### miRNA Discovery and mRNA Target Prediction in PPN

Identifying miRNAs in non-model organisms requires integrated approaches that combine computational prediction with experimental validation. To support this, a range of tools and databases have been developed. In this section, we review the key resources currently available to researchers investigating these questions in PPNs, as summarized in Table 2.

**Table 2:** Tools and databases commonly used in the study of non-model organisms’ miRNAs.

**Tool**	**Description**	**Access link**	**Reference**
**DATABASES**			
miRBase	The primary repository for miRNA sequences and annotation.	https://www.mirbase.org/	Kozomara et al. (2019)
MirGeneDB	High-confidence, manually curated miRNA gene database.	https://mirgenedb.org/	Clarke et al. (2025)
Rfam	Database of ncRNA families from a wide array of species.	https://rfam.org	Kalvari et al. (2021)
TargetWormScan	Searchable database of predicted regulatory targets of worm miRNAs.	https://www.targetscan.org/worm_52/	Lewis et al. (2005)
PmiREN	Comprehensive plant repository of plant miRNAs.	https://pmiren.com/	Guo et al. (2020)
ncPlantDB	Database specialized in ncRNAs in plants.	https://bis.zju.edu.cn/ncPlantDB/index/	Liu et al. (2025)
ExoCarta	Database containing information on exosomal proteins and RNAs including miRNAs.	http://exocarta.org/index.html	Keerthikumar et al. (2016)
miRTarBase	Comprehensive collection of validated miRNA-mRNA targets.	https://mirtarbase.cuhk.edu.cn/~miRTarBase/miRTarBase_2025/php/index.php	Cui et al. (2025)
miRecords	Resource for animal miRNA-target interactions including *C.elegans*.	http://c1.accurascience.com/miRecords/	Xiao et al. (2009)
**PREDICTION TOOLS**			
MirDeep2	Most widely used tool for both known and novel miRNA prediction in animals and plants.	https://github.com/rajewsky-lab/mirdeep2	Friedländer et al. (2012)
miRanalyzer	Tool for the detection of known, and prediction of new miRNAs, in high-throughput sequencing experiments.	http://bioinfo2.ugr.es/miRanalyzer/	Hackenberg et al. (2011)
miRPlant	miRNA predictor utilizing plant-specific parameters (e.g., handling of diverse hairpin lengths and sequences).	https://sourceforge.net/projects/mirplant/	An et al. (2014)
sRNAbench	Part of the sRNAToolKit suite for miRNA discovery and quantification using sRNASeq data.	http://bioinfo5.ugr.es/srnatoolbox/	Aparicio-Puerta et al. (2022)
ShortStack	Highly accurate, plant-optimized, and supports multi-mapping small RNAs.	https://github.com/MikeAxtell/ShortStac	Axtell (2013)
miRPara	Predicted miRNA precursors based on structural and sequence features.	https://github.com/weasteam/miRPara	Wu et al. (2011)
RNAFold	Comprehensive collection of tools for folding, design and analysis of RNA sequences.	http://rna.tbi.univie.ac.at/cgi-bin/RNAWebSuite/RNAfold.cgi	Gruber et al. (2008)
**TARGET PREDICTION**			
miRanda	An miRNA target scanner that aims to predict mRNA targets for miRNAs using dynamic-programming alignment and thermodynamics.	https://github.com/hacktrackgnulinux/miranda	Betel et al. (2010)
RNAHybrid	Tool for finding the minimum free energy hybridization of a long and a short RNA.	https://bibiserv.cebitec.uni-bielefeld.de/rnahybrid	Krüger and Rehmsmeier (2006)
psRNATarget	Specifically developed to identify target transcripts of plant regulatory sRNAs.	https://www.zhaolab.org/psRNATarget/	Dai and Zhao (2011)
p-TarPMir	Deep learning model adapted for plant miRNA target prediction.	https://ptarpmir.cu-bic.ca	Ajila et al. (2023b)
miTAR	Animal trained hybrid deep learning approach to predict miRNA targets.	https://github.com/tjgu/miTAR	Gu et al. (2021)

miRNAs, microRNAs; mRNA, messenger RNA.

The conserved nature of miRNA families across diverse organisms makes miRNA sequence databases powerful resources for identifying putative miRNAs in PPNs. By exploiting sequence homology with known miRNAs, such as those cataloged in miRBase, which includes over 38,000 unique miRNAs from various species, researchers have successfully identified candidate miRNAs in species like *H. glycines* and *M. incognita* (Subramanian et al., 2016; Lian et al., 2019). Importantly, the conserved nature of miRNA extends beyond sequence similarity; the regulatory functions of many miRNA families are also preserved across taxa, strengthening the rationale for cross-species prediction. Complementing this approach, databases such as TargetWormScan and miRTarBase provide extensive collections of computationally predicted and experimentally validated miRNA targets, offering valuable insights into the potential roles and functional relevance of these candidate miRNAs (Table 2).

Although database-driven discovery of miRNAs in PPNs provides a strong starting point, it is likely to miss species-specific miRNA families, an issue highlighted in *H. glycines,* with nearly half of its repertoire predicted as genus-specific (Ste-Croix et al., 2023). As a result, current best practices for miRNA studies recommend a combined approach: using database-guided predictions alongside *de novo* discovery methods based on sRNA deep-sequencing data (Yang and Li, 2011; Yang et al., 2021). Tools such as miRDeep2, miRAnalyser, and sRNABench (Table 2) can predict and quantify miRNAs using only sRNA sequencing data and homology-based training. However, their full potential is realized when genomic information, such as complete genomes or contig assemblies, is available alongside the sequencing data. The inclusion of genomic context allows for more accurate identification of precursor structures, improves the reliability of miRNA annotation, and enhances the prediction of novel miRNAs.

Nevertheless, while predicting miRNAs is an essential step, assigning them biological context and function is typically of greater interest. To this end, numerous tools have been developed for both model and non-model organisms to predict the targets of individual miRNA candidates. However, due to mechanistic differences in the machinery between plants and animals, selecting the appropriate prediction tool is crucial. For instance, tools like miRanda, an algorithm based on mammalian target recognition rules, can predict miRNA targets in nematodes but perform poorly in plant systems. It is also important to note that not all animal-trained algorithms perform equally well. For example, miTAR, which is trained on human data, performs worse in nematodes than miRanda, an algorithm partially trained on interactions identified in *C. elegans*. Similar considerations also apply to plant-specific predictors, which are typically optimized for plant-specific miRNA-mRNA interaction rules. Although Table 2 provides a useful starting point, more focused reviews on miRNA target prediction tools should be consulted when selecting an appropriate predictor (Carroll et al., 2014).

It is also worth noting the recent shift in PPN miRNA research toward machine learning-based and sequencing-free approaches. For instance, Ajila et al. (2023a) applied a retrained model based on the SMIRP framework, leveraging qualitative structural features, to identify species-specific miRNAs in *H. glycines*, predicting 3,342 pre-miRNA candidates. In the same study, machine learning was further employed to generate a species-specific model capable of predicting the *in vivo* targets of these miRNAs. Similar machine-learning strategies have also been implemented in plants and were recently reviewed by Jayasundara et al. (2021). While still in their early stages and prone to overestimating miRNA diversity, these approaches show great promise for expanding miRNA research across a broad range of species, requiring little more than a reference genome.

## Outlook and Future Directions

The field of miRNA research in relation to PPNs is rapidly evolving, with numerous avenues for future investigation. One promising direction is the exploration of miRNAs in regulating PPN pathogenicity and plant immune responses to nematodes. The potential for utilizing miRNA-based strategies, such as developing resistant plant varieties or synthetic miRNA decoys that disrupt regulatory networks, presents an exciting opportunity for agricultural biotechnology. While siRNAs have already demonstrated strong efficacy and remarkable specificity, miRNAs offer the added advantage of simultaneously targeting entire gene families, such as effector families, which could provide a more durable and broadly effective solution against PPN. Nonetheless, further research is essential to minimize potential off-target effects and ensure safe application.

To realize these applications, further research is needed to elucidate the complex regulatory networks involving miRNAs, their targets, and the signaling pathways they influence during PPN interactions. While computational predictions abound, few miRNA targets in PPNs have been experimentally validated. Techniques such as luciferase reporter assays, CRISPR-Cas9-mediated knockouts, and antisense oligonucleotides can provide direct evidence of miRNA function. Integrating these approaches with transcriptomic data across developmental stages will be essential to map the regulatory networks involved in parasitic success and life stage transitions. PPNs secrete hundreds of effectors to manipulate host cellular processes, yet little is known about how these genes are regulated at the post-transcriptional level. Given the temporal specificity of effector expression, miRNAs are strong candidates as regulators. Investigating whether specific miRNAs act as molecular switches to control effector expression during infection could reveal new aspects of PPN biology.

One of the most intriguing frontiers is the potential for cross-kingdom communication, wherein nematode miRNAs suppress host defense genes or, conversely, plant-derived miRNAs target nematode virulence factors. Experimental evidence for these interactions remains sparse. Future work should focus on validating predicted cross-kingdom miRNA targets using dual-host-pathogen expression systems and developing methods to trace miRNA movement between organisms. The pathways by which PPNs package and deliver miRNAs to host cells, possibly via EVs, also remain poorly understood. Identifying the proteins and signals involved in selective miRNA export, and determining whether this export occurs during specific stages, could open up new targets for nematode control or miRNA interception technologies.

In conclusion, the role of miRNAs in PPNs is a burgeoning area of research that holds significant promise for both basic and applied discoveries in plant-nematode interactions. Advancing this field will require interdisciplinary approaches and improved molecular tools to fully elucidate the role of miRNAs in PPNs. The potential for leveraging miRNAs in the development of new control strategies offers a hopeful avenue for mitigating the impact of PPNs on global agriculture.

## References

[j_jofnem-2025-0041_ref_001] Abbott A. L., Alvarez-Saavedra E., Miska E. A., Lau N. C., Bartel D. P., Horvitz H. R., Ambros V. (2005). The let-7 MicroRNA family members mir-48, mir-84, and mir-241 function together to regulate developmental timing in *Caenorhabditis elegans*. Developmental Cell.

[j_jofnem-2025-0041_ref_002] Ajila V., Colley L., Ste-Croix D. T., Nissan N., Cober E. R., Mimee B., Samanfar B., Green J. R. (2023a). Species-specific microRNA discovery and target prediction in the soybean cyst nematode. Scientific Reports.

[j_jofnem-2025-0041_ref_003] Ajila V., Colley L., Ste-Croix D. T., Nissan N., Golshani A., Cober E. R., Mimee B., Samanfar B., Green J. R. (2023b). P-TarPmiR accurately predicts plant-specific miRNA targets. Scientific Reports.

[j_jofnem-2025-0041_ref_004] An J., Lai J., Sajjanhar A., Lehman M. L., Nelson C. C. (2014). miRPlant: An integrated tool for identification of plant miRNA from RNA sequencing data. BMC Bioinformatics.

[j_jofnem-2025-0041_ref_005] Aparicio-Puerta E., Gómez-Martín C., Giannoukakos S., Medina J. M., Scheepbouwer C., García-Moreno A., Carmona-Saez P., Fromm B., Pegtel M., Keller A., Marchal J. A., Hackenberg M. (2022). sRNAbench and sRNAtoolbox 2022 update: Accurate miRNA and sncRNA profiling for model and non-model organisms. Nucleic Acids Research.

[j_jofnem-2025-0041_ref_006] Arroyo J. D., Chevillet J. R., Kroh E. M., Ruf I. K., Pritchard C. C., Gibson D. F., Mitchell P. S., Bennett C. F., Pogosova-Agadjanyan E. L., Stirewalt D. L., Tait J. F., Tewari M. (2011). Argonaute2 complexes carry a population of circulating microRNAs independent of vesicles in human plasma. Proceedings of the National Academy of Sciences of the United States of America.

[j_jofnem-2025-0041_ref_007] Axtell M. J. (2013). ShortStack: Comprehensive annotation and quantification of small RNA genes. RNA.

[j_jofnem-2025-0041_ref_008] Betel D., Koppal A., Agius P., Sander C., Leslie C. (2010). Comprehensive modeling of microRNA targets predicts functional non-conserved and non-canonical sites. Genome Biology.

[j_jofnem-2025-0041_ref_009] Bologna N. G., Iselin R., Abriata L. A., Sarazin A., Pumplin N., Jay F., Grentzinger T., Dal Peraro M., Voinnet O. (2018). Nucleo-cytosolic shuttling of ARGONAUTE1 prompts a revised model of the plant microRNA pathway. Molecular Cell.

[j_jofnem-2025-0041_ref_010] Brioudes F., Jay F., Sarazin A., Grentzinger T., Devers E. A., Voinnet O. (2021). HASTY, the *Arabidopsis* EXPORTIN5 ortholog, regulates cell-to-cell and vascular microRNA movement. The EMBO Journal.

[j_jofnem-2025-0041_ref_011] Buck A. H., Coakley G., Simbari F., McSorley H. J., Quintana J. F., Le Bihan T., Kumar S., Abreu-Goodger C., Lear M., Harcus Y., Ceroni A., Babayan S. A., Blaxter M., Ivens A., Maizels R. M. (2014). Exosomes secreted by nematode parasites transfer small RNAs to mammalian cells and modulate innate immunity. Nature Communications.

[j_jofnem-2025-0041_ref_012] Cai Q., He B., Wang S., Fletcher S., Niu D., Mitter N., Birch P. R. J., Jin H. (2021). Message in a bubble: Shuttling small RNAs and proteins between cells and interacting organisms using extracellular vesicles. Annual Review of Plant Biology.

[j_jofnem-2025-0041_ref_013] Carroll A. P., Goodall G. J., Liu B. (2014). Understanding principles of miRNA target recognition and function through integrated biological and bioinformatics approaches. Wiley Interdisciplinary Reviews RNA.

[j_jofnem-2025-0041_ref_014] Chen X., Liang H., Zhang J., Zen K., Zhang C.-Y. (2012). Secreted microRNAs: A new form of intercellular communication. Trends in Cell Biology.

[j_jofnem-2025-0041_ref_015] Chowdhury S., Sais D., Donnelly S., Tran N. (2024). The knowns and unknowns of helminth–host mirna cross-kingdom communication. Trends in Parasitology.

[j_jofnem-2025-0041_ref_016] Clarke A. W., Høye E., Hembrom A. A., Paynter V. M., Vinther J., Wyrożemski Ł, Biryukova I., Formaggioni A., Ovchinnikov V., Herlyn H., Pierce A., Wu C., Aslanzadeh M., Cheneby J., Martinez P., Friedländer M. R., Hovig E., Hackenberg M., Umu S. U., Johansen M., Peterson K. J., Fromm B. (2025). MirGeneDB 3.0: Improved taxonomic sampling, uniform nomenclature of novel conserved microRNA families and updated covariance models. Nucleic Acids Research.

[j_jofnem-2025-0041_ref_017] Cui S., Yu S., Huang H. Y., Lin Y. C. D., Huang Y., Zhang B., Xiao J., Zuo H., Wang J., Li Z., Li G., Ma J., Chen B., Zhang H., Fu J., Wang L., Huang H. D. (2025). miRTarBase 2025: Updates to the collection of experimentally validated microRNA–target interactions. Nucleic Acids Research.

[j_jofnem-2025-0041_ref_018] Dai X., Zhao P. X. (2011). psRNATarget: A plant small RNA target analysis server. Nucleic Acids Research.

[j_jofnem-2025-0041_ref_019] de Wit E., Linsen S. E. V., Cuppen E., Berezikov E. (2009). Repertoire and evolution of miRNA genes in four divergent nematode species. Genome Research.

[j_jofnem-2025-0041_ref_020] Ding X., Ye J., Wu X., Huang L., Zhu L., Lin S. (2015). Deep sequencing analyses of pine wood nematode *Bursaphelenchus xylophilus* microRNAs reveal distinct miRNA expression patterns during the pathological process of pine wilt disease. Gene.

[j_jofnem-2025-0041_ref_021] Dong Z., Han M.-H., Fedoroff N. (2008). The RNA-binding proteins HYL1 and SE promote accurate in vitro processing of pri-miRNA by DCL1. Proceedings of the National Academy of Sciences of the United States of America.

[j_jofnem-2025-0041_ref_022] Eves-van den Akker S. (2021). Plant–nematode interactions. Current Opinion in Plant Biology.

[j_jofnem-2025-0041_ref_023] Fabbri M., Paone A., Calore F., Galli R., Gaudio E., Santhanam R., Lovat F., Fadda P., Mao C., Nuovo G. J., Zanesi N., Crawford M., Ozer G. H., Wernicke D., Alder H., Caligiuri M. A., Nana-Sinkam P., Perrotti D., Croce C. M. (2012). MicroRNAs bind to Toll-like receptors to induce prometastatic inflammatory response. Proceedings of the National Academy of Sciences of the United States of America.

[j_jofnem-2025-0041_ref_024] Fire A., Xu S., Montgomery M. K., Kostas S. A., Driver S. E., Mello C. C. (1998). Potent and specific genetic interference by double-stranded RNA in *Caenorhabditis elegans*. Nature.

[j_jofnem-2025-0041_ref_025] Friedländer M. R., Mackowiak S. D., Li N., Chen W., Rajewsky N. (2012). miRDeep2 accurately identifies known and hundreds of novel microRNA genes in seven animal clades. Nucleic Acids Research.

[j_jofnem-2025-0041_ref_026] Gruber A. R., Lorenz R., Bernhart S. H., Neuböck R., Hofacker I. L. (2008). The Vienna RNA Websuite. Nucleic Acids Research.

[j_jofnem-2025-0041_ref_027] Gualtieri C., Leonetti P., Macovei A. (2020). Plant miRNA cross-kingdom transfer targeting parasitic and mutualistic organisms as a tool to advance modern agriculture. Frontiers in Plant Science.

[j_jofnem-2025-0041_ref_028] Gu T., Zhao X., Barbazuk W. B., Lee J.-H. (2021). miTAR: A hybrid deep learning-based approach for predicting miRNA targets. BMC Bioinformatics.

[j_jofnem-2025-0041_ref_029] Guo Z., Kuang Z., Wang Y., Zhao Y., Tao Y., Cheng C., Yang J., Lu X., Hao C., Wang T., Cao X., Wei J., Li L., Yang X. (2020). PmiREN: A comprehensive encyclopedia of plant miRNAs. Nucleic Acids Research.

[j_jofnem-2025-0041_ref_030] Hackenberg M., Rodríguez-Ezpeleta N., Aransay A. M. (2011). miRanalyzer: An update on the detection and analysis of microRNAs in high-throughput sequencing experiments. Nucleic Acids Research.

[j_jofnem-2025-0041_ref_031] He B., Wang H., Liu G., Chen A., Calvo A., Cai Q., Jin H. (2023). Fungal small RNAs ride in extracellular vesicles to enter plant cells through clathrin-mediated endocytosis. Nature Communications.

[j_jofnem-2025-0041_ref_032] Hewezi T. (2020). Epigenetic mechanisms in nematode–plant interactions. Annual Review of Phytopathology.

[j_jofnem-2025-0041_ref_033] Huang Q. X., Cheng X. Y., Mao Z. C., Wang Y. S., Zhao L. L., Yan X., Ferris V. R., Xu R. M., Xie B. Y. (2010). MicroRNA discovery and analysis of pinewood nematode *Bursaphelenchus xylophilus* by deep sequencing. PLoS ONE.

[j_jofnem-2025-0041_ref_034] Jaubert-Possamai S., Noureddine Y., Favery B. (2019). MicroRNAs, new players in the plant–nematode interaction. Frontiers in Plant Science.

[j_jofnem-2025-0041_ref_035] Jayasundara S., Lokuge S., Ihalagedara P., Herath D. (2021). Machine learning for plant microRNA prediction: A systematic review.

[j_jofnem-2025-0041_ref_036] Kalvari I., Nawrocki E. P., Ontiveros-Palacios N., Argasinska J., Lamkiewicz K., Marz M., Griffiths-Jones S., Toffano-Nioche C., Gautheret D., Weinberg Z., Rivas E., Eddy S. R., Finn R. D., Bateman A., Petrov A. I. (2021). Rfam 14: Expanded coverage of metagenomic, viral and microRNA families. Nucleic Acids Research.

[j_jofnem-2025-0041_ref_037] Kantor C., Eisenback J. D., Kantor M. (2024). Biosecurity risks to human food supply associated with plant-parasitic nematodes. Frontiers in Plant Science.

[j_jofnem-2025-0041_ref_038] Kaur P., Shukla N., Joshi G., VijayaKumar C., Jagannath A., Agarwal M., Goel S., Kumar A. (2017). Genome-wide identification and characterization of miRNAome from tomato (*Solanum lycopersicum*) roots and root-knot nematode (*Meloidogyne incognita*) during susceptible interaction. PLoS ONE.

[j_jofnem-2025-0041_ref_039] Keerthikumar S., Chisanga D., Ariyaratne D., Al Saffar H., Anand S., Zhao K., Samuel M., Pathan M., Jois M., Chilamkurti N., Gangoda L., Mathivanan S. (2016). ExoCarta: A web-based compendium of exosomal cargo. Journal of Molecular Biology.

[j_jofnem-2025-0041_ref_040] Koch L. (2014). easiRNAs—guardians of the plant genome. Nature Reviews Genetics.

[j_jofnem-2025-0041_ref_041] Kotagama K., McJunkin K. (2024). Recent advances in understanding microRNA function and regulation in *C. elegans*. Seminars in Cell and Developmental Biology.

[j_jofnem-2025-0041_ref_042] Kozomara A., Birgaoanu M., Griffiths-Jones S. (2019). miRBase: From microRNA sequences to function. Nucleic Acids Research.

[j_jofnem-2025-0041_ref_043] Krüger J., Rehmsmeier M. (2006). RNAhybrid: MicroRNA target prediction easy, fast and flexible. Nucleic Acids Research.

[j_jofnem-2025-0041_ref_044] Lee R. C., Feinbaum R. L., Ambros V. (1993). The *C. elegans* heterochronic gene *lin-4* encodes small RNAs with antisense complementarity to *lin-14*. Cell.

[j_jofnem-2025-0041_ref_045] Leonetti P., Dallera D., De Marchi D., Candito P., Pasotti L., Macovei A. (2024). Exploring the putative microRNAs cross-kingdom transfer in *Solanum lycopersicum-Meloidogyne incognita* interactions. Frontiers in Plant Science.

[j_jofnem-2025-0041_ref_046] Lewis B. P., Burge C. B., Bartel D. P. (2005). Conserved seed pairing, often flanked by adenosines, indicates that thousands of human genes are microRNA targets. Cell.

[j_jofnem-2025-0041_ref_047] Lian Y., Wei H., Wang J., Lei C., Li H., Li J., Wu Y., Wang S., Zhang H., Wang T., Du P., Guo J., Lu W. (2019). Chromosome-level reference genome of X12, a highly virulent race of the soybean cyst nematode *Heterodera glycines*. Molecular Ecology Resources.

[j_jofnem-2025-0041_ref_048] Liang H., Zen K., Zhang J., Zhang C. Y., Chen X. (2013). New roles for microRNAs in cross-species communication. RNA biology.

[j_jofnem-2025-0041_ref_049] Lin H., Spradling A. C. (1997). A novel group of pumilio mutations affects the asymmetric division of germline stem cells in the *Drosophila* ovary. Development.

[j_jofnem-2025-0041_ref_050] Liu H., Nichols R. L., Qiu L., Sun R., Zhang B., Pan X. (2019). Small RNA sequencing reveals regulatory roles of microRNAs in the development of *Meloidogyne incognita*. International Journal of Molecular Sciences.

[j_jofnem-2025-0041_ref_051] Liu L., Liu E., Hu Y., Li S., Zhang S., Chao H., Hu Y., Zhu Y., Chen Y., Xie L., Shen Y., Wu L., Chen M. (2025). ncPlantDB: A plant ncRNA database with potential ncPEP information and cell type-specific interaction. Nucleic Acids Research.

[j_jofnem-2025-0041_ref_052] Liu Y., Teng C., Xia R., Meyers B. C. (2020). PhasiRNAs in plants: Their biogenesis, genic sources, and roles in stress responses, development, and reproduction. The Plant Cell.

[j_jofnem-2025-0041_ref_053] Li J., Yang Z., Yu B., Liu J., Chen X. (2005). Methylation protects miRNAs and siRNAs from a 3′-end uridylation activity in *Arabidopsis*. Current Biology.

[j_jofnem-2025-0041_ref_054] Mallory A., Vaucheret H. (2010). Form, function, and regulation of Argonaute proteins. The Plant Cell.

[j_jofnem-2025-0041_ref_055] Mani V., Assefa A. D., Hahn B.-S. (2021). Transcriptome analysis and miRNA target profiling at various stages of root-knot nematode *Meloidogyne incognita* development for identification of potential regulatory networks. International Journal of Molecular Sciences.

[j_jofnem-2025-0041_ref_056] Marks N. D., Winter A. D., Gu H. Y., Maitland K., Gillan V., Ambroz M., Martinelli A., Laing R., MacLellan R., Towne J., Roberts B., Hanks E., Devaney E., Britton C. (2019). Profiling microRNAs through development of the parasitic nematode *Haemonchus* identifies nematode-specific miRNAs that suppress larval development. Scientific Reports.

[j_jofnem-2025-0041_ref_057] Meng X., Jin W., Wu F. (2020). Novel tomato miRNA miR1001 initiates cross-species regulation to suppress the conidiospore germination and infection virulence of *Botrytis cinerea* in vitro. Gene.

[j_jofnem-2025-0041_ref_058] O’Brien J., Hayder H., Zayed Y., Peng C. (2018). Overview of microRNA biogenesis, mechanisms of actions, and circulation. Frontiers in Endocrinology.

[j_jofnem-2025-0041_ref_059] Piatek M. J., Werner A. (2014). Endogenous siRNAs: Regulators of internal affairs. Biochemical Society Transactions.

[j_jofnem-2025-0041_ref_060] Rambani A., Hu Y., Piya S., Long M., Rice J. H., Pantalone V., Hewezi T. (2020). Identification of differentially methylated miRNA genes during compatible and incompatible interactions between soybean and soybean cyst nematode. Molecular Plant-Microbe Interactions: MPMI.

[j_jofnem-2025-0041_ref_061] Reinhart B. J., Slack F. J., Basson M., Pasquinelli A. E., Bettinger J. C., Rougvie A. E., Horvitz H. R., Ruvkun G. (2000). The 21-nucleotide let-7 RNA regulates developmental timing in *Caenorhabditis elegans*. Nature.

[j_jofnem-2025-0041_ref_062] Rogers K., Chen X. (2013). Biogenesis, turnover, and mode of action of plant microRNAs. The Plant Cell.

[j_jofnem-2025-0041_ref_063] Santangelo L., Giurato G., Cicchini C., Montaldo C., Mancone C., Tarallo R., Battistelli C., Alonzi T., Weisz A., Tripodi M. (2016). The RNA-binding protein SYNCRIP is a component of the hepatocyte exosomal machinery controlling microRNA sorting. Cell Reports.

[j_jofnem-2025-0041_ref_064] Siddique S., Grundler F. M. (2018). Parasitic nematodes manipulate plant development to establish feeding sites. Current Opinion in Microbiology.

[j_jofnem-2025-0041_ref_065] Song L., Axtell M. J., Fedoroff N. V. (2010). RNA secondary structural determinants of miRNA precursor processing in *Arabidopsis*. Current Biology.

[j_jofnem-2025-0041_ref_066] Ste-Croix D. T., Bélanger R. R., Mimee B. (2023). Characterization of microRNAs in the cyst nematode *Heterodera glycines* identifies possible candidates involved in cross-kingdom interactions with its host *Glycine max*. RNA Biology.

[j_jofnem-2025-0041_ref_067] Subramanian P., Choi I. C., Mani V., Park J., Subramaniyam S., Choi K. H., Sim J. S., Lee C. M., Koo J. C., Hahn B. S. (2016). Stage-wise identification and analysis of miRNA from root-knot nematode *Meloidogyne incognita*. International Journal of Molecular Sciences.

[j_jofnem-2025-0041_ref_068] Sun Y. H., Lee B., Li X. Z. (2022). The birth of piRNAs: How mammalian piRNAs are produced, originated, and evolved. Mammalian Genome.

[j_jofnem-2025-0041_ref_069] Tian Z., Cai Y., Zhu M., Wang L., Liu Q., Li Q., Gao X., Zheng J., Lin B., Zhuo K., Han S. (2025). mgr-mir-228-regulated transthyretin-like protein in *Meloidogyne graminicola* suppresses ROS generation and enhances parasitism. Phytopathology Research.

[j_jofnem-2025-0041_ref_070] Tian Z., Zhou J., Zheng J., Han S. (2023). mgr-mir-9 implicates *Meloidogyne graminicola* infection in rice by targeting the effector *MgPDI*. Journal of Integrative Agriculture.

[j_jofnem-2025-0041_ref_071] Turchinovich A., Samatov T. R., Tonevitsky A. G., Burwinkel B. (2013). Circulating miRNAs: Cell–cell communication function?. Frontiers in Genetics.

[j_jofnem-2025-0041_ref_072] Turchinovich A., Weiz L., Langheinz A., Burwinkel B. (2011). Characterization of extracellular circulating microRNA. Nucleic Acids Research.

[j_jofnem-2025-0041_ref_073] Vieira P., Gleason C. (2019). Plant-parasitic nematode effectors–insights into their diversity and new tools for their identification. Current Opinion in Plant Biology.

[j_jofnem-2025-0041_ref_074] Wagner J., Riwanto M., Besler C., Knau A., Fichtlscherer S., Röxe T., Zeiher A. M., Landmesser U., Dimmeler S. (2013). Characterization of levels and cellular transfer of circulating lipoprotein-bound microRNAs. Arteriosclerosis, Thrombosis, and Vascular Biology.

[j_jofnem-2025-0041_ref_075] Wang B., Sun Y., Song N., Zhao M., Liu R., Feng H., Wang X., Kang Z. (2017). *Puccinia striiformis* f. sp. *tritici* microRNA-like RNA 1 (Pst-milR1), an important pathogenicity factor of Pst, impairs wheat resistance to Pst by suppressing the wheat pathogenesis-related 2 gene. The New Phytologist.

[j_jofnem-2025-0041_ref_076] Wang J., Mei J., Ren G. (2019). Plant microRNAs: Biogenesis, homeostasis, and degradation. Frontiers in Plant Science.

[j_jofnem-2025-0041_ref_077] Wang W., Liu D., Zhang X., Chen D., Cheng Y., Shen F. (2018). Plant microRNAs in cross-kingdom regulation of gene expression. International Journal of Molecular Sciences.

[j_jofnem-2025-0041_ref_078] Wang Y., Mao Z., Yan J., Cheng X., Liu F., Xiao L., Dai L., Luo F., Xie B. (2015). Identification of microRNAs in *Meloidogyne incognita* using deep sequencing. PLoS ONE.

[j_jofnem-2025-0041_ref_079] Weiberg A., Wang M., Lin F.-M., Zhao H., Zhang Z., Kaloshian I., Huang H.-D., Jin H. (2013). Fungal small RNAs suppress plant immunity by hijacking host RNA interference pathways. Science.

[j_jofnem-2025-0041_ref_080] Wightman B., Ha I., Ruvkun G. (1993). Posttranscriptional regulation of the heterochronic gene *lin-14* by *lin-4* mediates temporal pattern formation in *C. elegans*. Cell.

[j_jofnem-2025-0041_ref_081] Wu F., Huang Y., Jiang W., Jin W. (2023). Genome-wide identification and validation of tomato-encoded sRNA as the cross-species antifungal factors targeting the virulence genes of *Botrytis cinerea*. Frontiers in Plant Science.

[j_jofnem-2025-0041_ref_082] Wu Y., Wei B., Liu H., Li T., Rayner S. (2011). MiRPara: A SVM-based software tool for prediction of most probable microRNA coding regions in genome scale sequences. BMC Bioinformatics.

[j_jofnem-2025-0041_ref_083] Xiao F., Zuo Z., Cai G., Kang S., Gao X., Li T. (2009). miRecords: An integrated resource for microRNA-target interactions. Nucleic Acids Research.

[j_jofnem-2025-0041_ref_084] Xu J., Chen Q., Zen K., Zhang C., Zhang Q. (2013). Synaptosomes secrete and uptake functionally active microRNAs via exocytosis and endocytosis pathways. Journal of Neurochemistry.

[j_jofnem-2025-0041_ref_085] Yang X., Fishilevich E., German M. A., Gandra P., McEwan R. E., Billion A., Knorr E., Vilcinskas A., Narva K. E. (2021). Elucidation of the microRNA transcriptome in western corn rootworm reveals its dynamic and evolutionary complexity. Genomics, Proteomics and Bioinformatics.

[j_jofnem-2025-0041_ref_086] Yang X., Li L. (2011). miRDeep-P: A computational tool for analyzing the microRNA transcriptome in plants. Bioinformatics (Oxford, England).

[j_jofnem-2025-0041_ref_087] Yoshikawa M. (2013). Biogenesis of trans-acting siRNAs, endogenous secondary siRNAs in plants. Genes and Genetic Systems.

[j_jofnem-2025-0041_ref_088] Zhang J., Li S., Li L., Li M., Guo C., Yao J., Mi S. (2015). Exosome and exosomal microRNA: Trafficking, sorting, and function. Genomics, Proteomics and Bioinformatics.

[j_jofnem-2025-0041_ref_089] Zhang L., Hou D., Chen X., Li D., Zhu L., Zhang Y., Li J., Bian Z., Liang X., Cai X., Yin Y., Wang C., Zhang T., Zhu D., Zhang D., Xu J., Chen Q., Ba Y., Liu J., Wang Q., Chen J., Wang J., Wang M., Zhang Q., Zhang J., Zen K., Zhang C.-Y. (2012). Exogenous plant MIR168a specifically targets mammalian LDLRAP1: Evidence of cross-kingdom regulation by microRNA. Cell Research.

[j_jofnem-2025-0041_ref_090] Zhang T., Zhao Y. L., Zhao J. H., Wang S., Jin Y., Chen Z. Q., Fang Y. Y., Hua C. L., Ding S. W., Guo H. S. (2016a). Cotton plants export microRNAs to inhibit virulence gene expression in a fungal pathogen. Nature Plants.

[j_jofnem-2025-0041_ref_091] Zhang Y., Wang Y., Xie F., Li C., Zhang B., Nichols R. L., Pan X. (2016b). Identification and characterization of microRNAs in the plant parasitic root-knot nematode *Meloidogyne incognita* using deep sequencing. Functional and Integrative Genomics.

[j_jofnem-2025-0041_ref_092] Zhao C., Sun X., Li L. (2019). Biogenesis and function of extracellular miRNAs. ExRNA.

[j_jofnem-2025-0041_ref_093] Zhou G., Zhou Y., Chen X. (2017). New insight into inter-kingdom communication: horizontal transfer of mobile small RNAs. Frontiers in microbiology.

